# Isolated Abdominal Wall Metastasis of Hepatocellular Carcinoma: A Case Report

**DOI:** 10.7759/cureus.102707

**Published:** 2026-01-31

**Authors:** Sofia Sequeira, Ana Silva, Inês Peneda Ferreira, Sónia Carvalho, José Presa Ramos

**Affiliations:** 1 Internal Medicine, Hospital de Santo Espírito da Ilha Terceira, Angra do Heroísmo, PRT; 2 Internal Medicine, Unidade Local de Saúde de Gaia e Espinho, Vila Nova de Gaia, PRT; 3 Internal Medicine, Unidade Local de Saúde de Trás-os-Montes e Alto Douro, Vila Real, PRT

**Keywords:** abdominal wall metastasis, chronic hepatitis b, extrahepatic metastasis, hepatocellular carcinoma, surgical excision

## Abstract

Hepatocellular carcinoma (HCC) is the most common primary malignancy of the liver and typically develops in the setting of chronic liver disease. Extrahepatic metastases usually occur in advanced disease stages and most frequently involve the lungs, bones, and lymph nodes, while metastasis to the abdominal wall is exceedingly rare. We report the case of a 74-year-old woman with chronic hepatitis B-related liver disease who was previously treated with curative-intent hepatectomy and later underwent percutaneous ablation for intrahepatic tumor recurrence. During routine surveillance, a solitary lesion of the right anterolateral abdominal wall was detected and histologically confirmed as metastatic HCC. Importantly, the lesion was anatomically distant from all prior surgical and percutaneous access sites, effectively excluding procedure-related tumor seeding as a mechanism of spread. Comprehensive staging revealed no additional metastatic disease, and the lesion was surgically excised. This case highlights an unusual pattern of extrahepatic dissemination of HCC and emphasizes the importance of careful anatomical assessment and long-term surveillance for the early detection of atypical metastatic disease.

## Introduction

Hepatocellular carcinoma (HCC) accounts for the majority of primary liver cancers and represents a major global health burden [1]. It most commonly arises in patients with chronic liver disease, particularly cirrhosis related to viral hepatitis, alcohol misuse, or metabolic-associated fatty liver disease [2].

HCC characteristically spreads via vascular invasion and intrahepatic dissemination. Extrahepatic metastases are less common at diagnosis but may occur during disease progression, most frequently affecting the lungs, bones, lymph nodes, and adrenal glands [3]. Metastases to the abdominal wall are exceptionally rare and, when present, are typically attributed to direct implantation along surgical or percutaneous intervention tracts [4].

We describe a rare case of isolated abdominal wall metastasis of HCC occurring years after curative-intent treatment, with no anatomical relationship to previous percutaneous access routes, underscoring an unusual metastatic pattern.

## Case presentation

A 74-year-old woman with advanced chronic liver disease secondary to chronic hepatitis B infection was diagnosed with early-stage HCC (Barcelona Clinic Liver Cancer stage 0) located in segment II during routine imaging surveillance. She was under antiviral therapy with entecavir 0.5 mg daily, with undetectable hepatitis B virus DNA levels.

At baseline, liver disease was clinically compensated, with preserved synthetic function, including normal total bilirubin, albumin, and international normalized ratio, consistent with Child-Pugh class A. Transient elastography demonstrated liver stiffness compatible with stage F2 hepatic fibrosis (10.7 kPa), with a controlled attenuation parameter of 162 dB/m. Despite preserved liver function, features of clinically significant portal hypertension were present, including grade I esophageal varices identified on upper endoscopy and a prior history of therapeutic paracentesis. Platelet count remained above 100×10³/L.

The patient underwent atypical hepatectomy with curative intent.

Two years later, follow-up revealed elevated alpha-fetoprotein levels (187 IU/mL; normal range: 1-8 IU/mL). Abdominal computed tomography (CT) identified a new subcapsular nodular lesion at the junction of segments V and VI. Percutaneous biopsy confirmed recurrent HCC. Surgical resection was proposed but declined by the patient, and percutaneous thermal ablation was performed.

**Figure 1:**
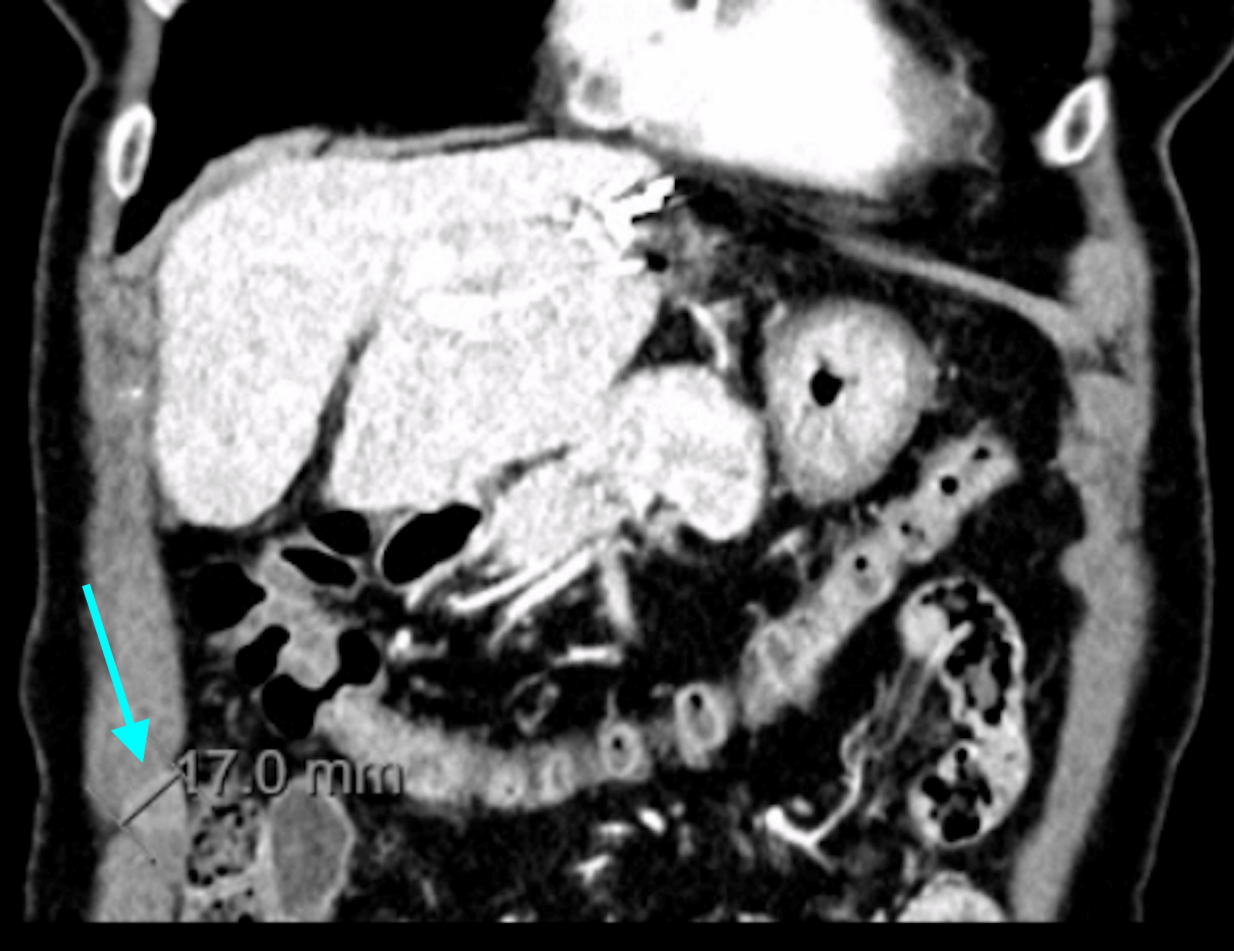
Contrast-enhanced abdominal computed tomography demonstrating a nodular lesion within the right anterolateral abdominal wall musculature, measuring approximately 17 mm, with heterogeneous enhancement and central washout in delayed phases, suggestive of malignancy

One year after ablation, surveillance CT demonstrated a new 17 mm nodular lesion within the right anterolateral abdominal wall musculature, exhibiting heterogeneous enhancement and central washout on delayed phases, raising suspicion of malignancy. There was no evidence of recurrence at the ablation site or elsewhere in the liver (Figure 1).

Laboratory evaluation at the time of detection of the abdominal wall lesion showed mildly elevated alpha-fetoprotein levels (46.4 IU/mL) with preserved liver function, including normal aminotransferases, bilirubin, albumin, and international normalized ratio, and a platelet count of 136×10³/L (Table 1).

**Table 1:** Laboratory findings at the time of detection of the abdominal wall lesion

Parameter	Result	Reference range
Alpha-fetoprotein (AFP)	46.4 IU/mL	1-8 IU/mL
Aspartate aminotransferase (AST)	24 U/L	<35 U/L
Alanine aminotransferase (ALT)	22 U/L	<33 U/L
Total bilirubin	0.7 mg/dL	<1.2 mg/dL
Albumin	4.4 g/dL	3.4-4.8 g/dL
International normalized ratio (INR)	1.13	<1.2
Platelets	136×10³/L	150-400×10³/L

**Figure 2:**
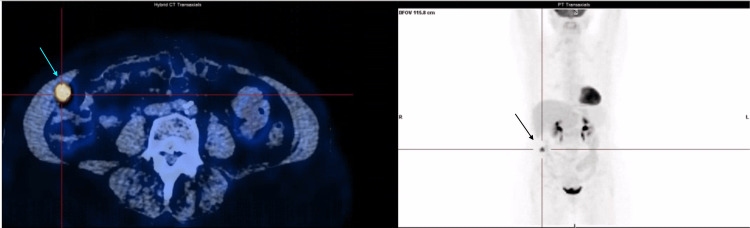
Positron emission tomography-computed tomography showing a solitary hypermetabolic lesion in the right anterolateral abdominal wall, consistent with isolated metastatic disease, with no evidence of additional hypermetabolic foci

Ultrasound-guided biopsy of the abdominal wall lesion confirmed metastatic HCC. Given the unusual presentation of a solitary soft tissue metastasis, comprehensive staging was performed using positron emission tomography-computed tomography (PET-CT), which demonstrated a single hypermetabolic lesion confined to the right anterolateral abdominal wall, with no evidence of additional intrahepatic or extrahepatic disease (Figure 2).

Following multidisciplinary discussion, surgical excision of the lesion was performed. Histopathological analysis confirmed metastatic HCC. The metastatic focus was anatomically distant from both the surgical field and the percutaneous ablation access site, with no spatial or anatomical continuity, effectively excluding needle-track seeding. In the absence of additional metastatic disease, active surveillance was recommended, with follow-up PET-CT scheduled three months later.

## Discussion

Extrahepatic dissemination of HCC is typically associated with advanced tumor stage, vascular invasion, and poor prognosis, most commonly involving the lungs, bones, lymph nodes, and adrenal glands [3,5]. In contrast, metastatic involvement of the abdominal wall is exceedingly rare and, when reported, is most often attributed to iatrogenic tumor implantation following diagnostic or therapeutic percutaneous procedures, such as liver biopsy or thermal ablation [4,6].

Needle-track seeding is a recognized but uncommon complication in the management of HCC, with reported incidence rates ranging from 0.6% to 5%, depending on tumor characteristics and procedural technique [6]. Such metastases usually arise along the direct anatomical trajectory of the needle and are frequently associated with local tumor recurrence at the treated hepatic site [4,6]. Consequently, the detection of an abdominal wall mass in a patient with prior percutaneous hepatic interventions typically raises concern for procedure-related tumor seeding.

However, in the present case, several clinical, radiological, and anatomical features strongly argue against this mechanism. Specifically, the percutaneous thermal ablation was performed through a right subcostal approach, with the needle trajectory anatomically confined to the upper abdominal wall and clearly distant from the right anterolateral abdominal wall musculature where the metastatic lesion subsequently developed. There was no spatial continuity between the ablation tract and the site of the abdominal wall metastasis, nor was there imaging or clinical evidence of tumor implantation along the needle path during follow-up. Furthermore, imaging surveillance demonstrated complete local control at the ablation site, with no evidence of residual or recurrent intrahepatic disease at that location.

The isolated nature of the metastatic lesion further supports an alternative mechanism of spread. Comprehensive staging with PET-CT confirmed the absence of additional intrahepatic or extrahepatic disease, an unusual finding in HCC, which typically demonstrates multifocal or systemic dissemination when extrahepatic spread occurs [3,5]. The delayed temporal presentation and solitary soft tissue involvement, in the absence of peritoneal disease, are more consistent with hematogenous dissemination than with direct implantation.

The biological mechanisms underlying atypical metastatic patterns in HCC remain incompletely understood. Hematogenous spread through the systemic circulation, potentially facilitated by microscopic vascular invasion or circulating tumor cells, may allow tumor implantation in distant and uncommon sites even in patients initially diagnosed with early-stage disease and treated with curative intent [7]. This case illustrates that early-stage HCC and preserved liver function do not entirely preclude late and unpredictable metastatic behavior.

From a clinical perspective, this case underscores the importance of maintaining a high index of suspicion during the long-term surveillance of patients with HCC. Abdominal wall lesions detected during follow-up should not be automatically attributed to procedure-related tumor seeding, particularly when they are anatomically remote from previous surgical or percutaneous access sites. Careful anatomical correlation, histopathological confirmation, and comprehensive staging are essential to establish the correct diagnosis and guide management.

Importantly, the identification of a solitary extrahepatic metastasis in this patient enabled curative-intent surgical excision. Available evidence suggests that selected patients with controlled intrahepatic disease, preserved liver function, and isolated extrahepatic metastases may benefit from surgical resection, achieving prolonged survival compared with non-surgical management [8]. This highlights the value of a multidisciplinary approach and individualized decision-making in rare metastatic presentations of HCC.

Limitations

This report describes a single clinical case, which inherently limits the ability to generalize the findings. While the observations provide relevant clinical insights, they may not be applicable to all patients or clinical settings. Further studies with larger sample sizes are needed to better characterize this condition and its management.

## Conclusions

Isolated abdominal wall metastasis from HCC is an exceptionally rare manifestation of extrahepatic spread. This case demonstrates that abdominal wall lesions detected during follow-up should not be automatically attributed to procedure-related tumor seeding, particularly when they are anatomically distant from prior surgical or percutaneous access sites. Careful anatomical correlation, histopathological confirmation, and comprehensive staging are essential to establish the correct diagnosis.

Furthermore, the recognition of a solitary extrahepatic metastasis may allow for curative-intent local treatment in selected patients with preserved liver function and controlled intrahepatic disease. This case reinforces the importance of long-term surveillance and a multidisciplinary approach in the management of HCC, even in patients initially treated with curative intent.

## References

[1] Llovet JM, Kelley RK, Villanueva A (2021). Hepatocellular carcinoma. Nat Rev Dis Primers.

[2] Forner A, Reig M, Bruix J (2018). Hepatocellular carcinoma. Lancet.

[3] Katyal S, Oliver JH 3rd, Peterson MS, Ferris JV, Carr BS, Baron RL (2000). Extrahepatic metastases of hepatocellular carcinoma. Radiology.

[4] Chang S, Kim SH, Lim HK (2008). Needle tract implantation after percutaneous interventional procedures in hepatocellular carcinomas: lessons learned from a 10-year experience. Korean J Radiol.

[5] Uka K, Aikata H, Takaki S (2007). Clinical features and prognosis of patients with extrahepatic metastases from hepatocellular carcinoma. World J Gastroenterol.

[6] Silva MA, Hegab B, Hyde C, Guo B, Buckels JA, Mirza DF (2008). Needle track seeding following biopsy of liver lesions in the diagnosis of hepatocellular cancer: a systematic review and meta-analysis. Gut.

[7] Yang JD, Roberts LR (2010). Hepatocellular carcinoma: a global view. Nat Rev Gastroenterol Hepatol.

[8] Chan KM, Yu MC, Wu TJ, Lee CF, Chen TC, Lee WC, Chen MF (2009). Efficacy of surgical resection in management of isolated extrahepatic metastases of hepatocellular carcinoma. World J Gastroenterol.

